# Infectious Bronchitis Coronavirus Infection in Chickens: Multiple System Disease with Immune Suppression

**DOI:** 10.3390/pathogens9100779

**Published:** 2020-09-24

**Authors:** Shahnas M. Najimudeen, Mohamed S. H. Hassan, Susan C. Cork, Mohamed Faizal Abdul-Careem

**Affiliations:** Department of Ecosystem and Public Health, Faculty of Veterinary Medicine, University of Calgary, Health Research Innovation Center 2C53, 3330 Hospital Drive NW, Calgary, AB T2N 4N1, Canada; fathimashahnas.moham@ucalgary.ca (S.M.N.); msh.hassan@ucalgary.ca (M.S.H.H.); sccork@ucalgary.ca (S.C.C.)

**Keywords:** infectious bronchitis coronavirus, pathogenesis, tissue tropism, molecular epidemiology, chicken

## Abstract

In the early 1930s, infectious bronchitis (IB) was first characterized as a respiratory disease in young chickens; later, the disease was also described in older chickens. The etiology of IB was confirmed later as being due to a coronavirus: the infectious bronchitis virus (IBV). Being a coronavirus, IBV is subject to constant genome change due to mutation and recombination, with the consequence of changing clinical and pathological manifestations. The potential use of live attenuated vaccines for the control of IBV infection was demonstrated in the early 1950s, but vaccine breaks occurred due to the emergence of new IBV serotypes. Over the years, various IBV genotypes associated with reproductive, renal, gastrointestinal, muscular and immunosuppressive manifestations have emerged. IBV causes considerable economic impacts on global poultry production due to its pathogenesis involving multiple body systems and immune suppression; hence, there is a need to better understand the pathogenesis of infection and the immune response in order to help developing better management strategies. The evolution of new strains of IBV during the last nine decades against vaccine-induced immune response and changing clinical and pathological manifestations emphasize the necessity of the rational development of intervention strategies based on a thorough understanding of IBV interaction with the host.

## 1. Introduction

Infectious bronchitis virus (IBV) belongs to the family *Coronaviridae* and order *Nidovirales* [1]. IBV infects chickens and pheasants and induces a clinical disease known as infectious bronchitis (IB) [2]. The infected chickens may appear depressed with various levels of breathing difficulty and have ruffled feathers [3,4,5,6]. Younger chickens show the most severe clinical manifestations [7]. In addition, there is serological evidence that poultry workers may develop anti-IBV antibodies following exposure to infected birds, although there is no evidence of active infection in humans [8]. However, experimentally, certain IBV strains (i.e., Massachusetts (Mass) and Gray) have been found to be capable of replicating in human cell lines [9].

The first IB case caused by the Mass serotype was recorded from North Dakota, USA [10]. Since then, hundreds of IBVs with heterogenous genomes have been emerging continuously as a result of mutations and recombination [11,12]. Consequently, high IB-associated losses are recorded in spite of control attempts using live attenuated vaccines. Different strains of IBV demonstrate varying properties with differences in pathogenesis, virulence, tissue and age tropisms and receptor specificities [13,14,15,16,17]. Therefore, knowledge of different IBV strains and their pathogenicity is important for establishing sustainable vaccination strategies. Despite several published reviews relevant to IBV pathogenesis [18,19,20,21,22], there is a need for an analysis of information due to the large influx of literature in recent years on the evolving pathogenesis of IBV infection [23,24,25].

## 2. IBV Genetic Heterogeneity and Tissue Tropism

The variability of the spike (S)1 amino acid sequence determines the host and tissue tropism of IBV [26,27] since it engages the host cell receptor, α 2,3-linked sialic acids and/or heparan sulfate for entry [28,29]. It has been indicated that IBV’s ability to infect certain tissues is regulated by the avidity of S1 to α 2,3-linked sialic acids [30]. This avidity, in turn, is influenced by the glycosylation of the receptor-binding domain of the S1 [31]. However, it is not certain that these two receptors are exclusively engaged in IBV host cell entry, and the potential existence of an uncharacterized receptor has been suggested [17].

**Figure 1:**
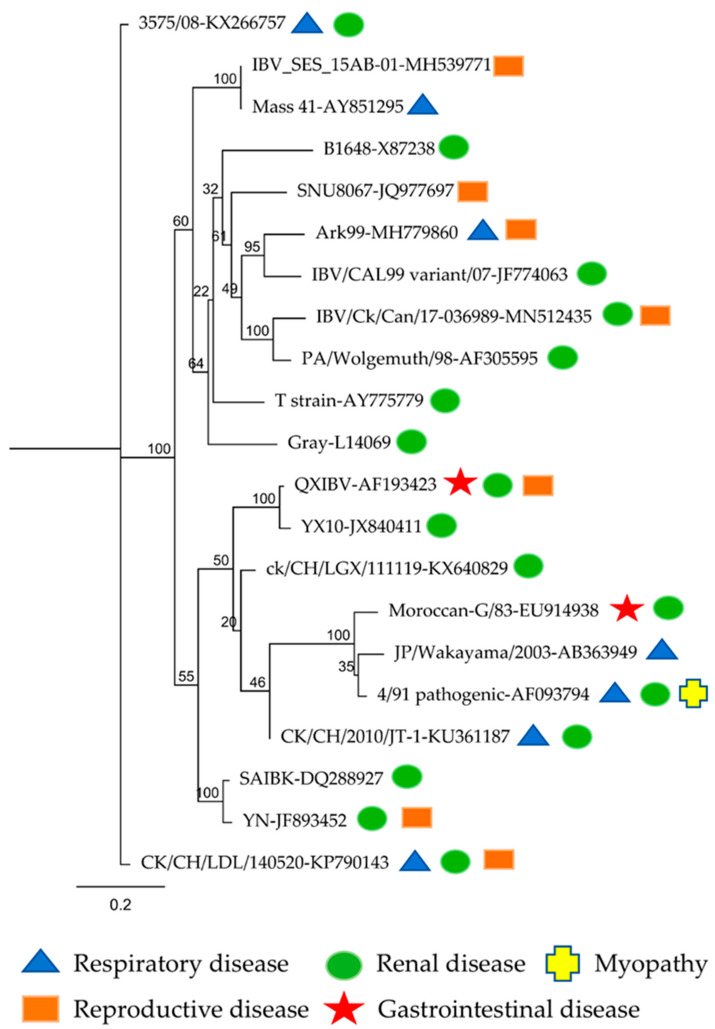
Phylogenetic tree based on the S1 nucleotide sequences (from the ATG start codon to the cleavage site of the spike protein). The phylogeny contains a total of 21 infectious bronchitis virus (IBV) strains from different countries around the world. The IBV strain and GenBank accession number are given for each strain. The sequences were aligned using Clustal Omega, and the phylogenetic tree was constructed using the maximum likelihood method available in RAxML with 1000 bootstrap replicates for branch support (the numbers on the nodes represent the bootstrap values). The analyses were constructed using Geneious^®^ v10.2.6 (https://www.geneious.com/).

The evolution and occurrence of novel strains of IBV is a continuing process [11]. Although the primary site of infection for most IBV strains was found to be the respiratory tract, the primary direction has been changing, involving multiple body systems with the rise of novel IBV variants [32,33,34,35,36,37]. A summary of the primary tissue tropism of different IBV strains in chickens is presented in Figure 1. Reproductive tract defects following infection with some IBV strains were first reported by van Roekel et al. in 1951, and the involved IBV serotype was Mass [36]. Cumming originally recorded the IBV strain pathogenic to kidney, Australian T (Aust T), in 1962 [38,39]. The 4/91 strain of IBV has been known to spread to muscle tissues and lead to bilateral myopathy [34]. In addition to these tissues, IBV targets gastrointestinal tissues without leading to pathological manifestations [35]. Kidney and cecal tonsils can harbor certain IBV strains for weeks [3,40]. The recently described IBV strains TW-like GD strain, Egypt/Beni-Suef/01 and QX-like strain also have a predilection for cells in immune organs, such as the bursa of Fabricius, cecal tonsils, spleen and Harderian gland [41,42,43,44]. In addition to the different virulence levels of the IBV strains, factors such as the type of host, vaccination history or immunocompetence, age of the host and environmental conditions determine the pathogenicity and severity of IB [15,32,45]. 

## 3. Establishment of Initial IBV Infection

IBV is typically transmitted to the host by inhalation, whereupon it attaches to the respiratory epithelium and enters by receptor-mediated endocytosis [46]. Once IBV enters the upper respiratory tract, it targets the epithelium, which is ciliated and includes mucus-secreting glands [30], causing ciliostasis and mucus accumulation. The virus replication in the respiratory epithelium peaks 3–7 days post-infection (dpi) depending on the infecting IBV strain [35,47,48]. Consequently, respiratory signs result within 2 dpi and peak around 6 dpi [49,50,51]. These respiratory clinical manifestations include sneezing, gasping, coughing, tracheal rales, nasal discharge, conjunctivitis, and dyspnea [3]. It is not uncommon to observe non-respiratory clinical manifestations such as depression, weight loss, lethargy and huddling together [32,50,52]. The morbidity and mortality associated with respiratory tract IBV infection depend on the age of infection; young chickens are severely affected compared to adult chickens [7,53]. At post-mortem examination, hemorrhages and the accumulation of caseous, serous and catarrhal exudates in the trachea, nasal passage and sinuses [47,52] are evident, as well as gross changes in air sacs (i.e., the accumulation of foamy or cloudy exudates) [54]. Depending on the time of sampling, histological features include deciliation and dislodgment of epithelial cells, as well as mononuclear cell infiltration. These changes are visible around 2–3 dpi. Further development of the lesions, with hyperplasia and hypertrophy of the epithelium and prominent mononuclear cell infiltration in lamina propria, are visible around 4–6 dpi. These stages are followed by recovery with repopulation of the mucosa with pseudostratified ciliated epithelium and goblet cells (10–20 dpi) [47,55,56].

## 4. Mechanisms of IBV Dissemination Beyond the Respiratory System

**Figure 2:**
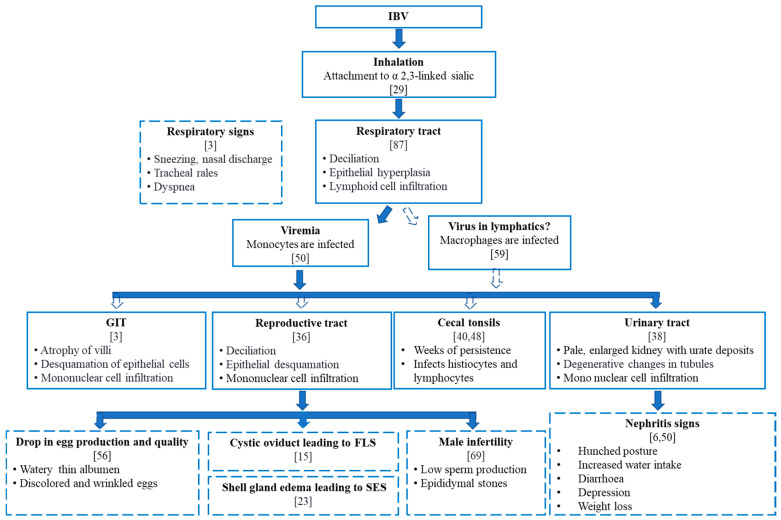
Clinical and pathological manifestations of IBV infection. The schematic diagram shows the entry, the route of dissemination of the virus to visceral organs and the pathogenesis of various IBV strains in different body systems. All the IBV strains primarily infect the respiratory tract, and based on the genotypes, the IBV infection can extend to various tissues, either persisting or leading to clinical and pathological manifestations. The solid arrows indicate paths that have been confirmed. The empty arrows indicate paths that have been suggested. The text boxes with continuous borders summarize histological changes, and the text boxes with discontinuous borders represent clinical manifestations. SES, shell-less egg syndrome; FLS, false layer syndrome; GIT, gastrointestinal tract.

It is well established that, following the initial infection in the respiratory tract, the virus is disseminated to other tissues due to viremia [18,57] (Figure 2). However, the exact mechanisms by which IBV leads to viremia are not understood. Recently, Reddy et al. (2016) showed that a Belgian nephropathogenic IBV strain, B1648, could infect blood monocytes and that these monocytes may facilitate the dissemination of IBV to visceral organs, including the kidney [50]. In agreement with this finding, another study [58] showed tropism of IBV towards monocytes by the Mass 41, California (Cal)99, Connecticut (Conn)46 and Iowa97 IBV strains. Using the Mass and Conn IBV strains, Amarasinghe et al. (2017) showed that IBV could infect low numbers of respiratory tract macrophages [59]. It is also possible that IBV dissemination beyond the respiratory tract may involve the lymphatic system and infected macrophages, similar to Marek’s disease virus dissemination via infected lung macrophages [60]. 

## 5. IBV Infection of the Reproductive System

Subsequent clinical manifestations of IBV infection related to the reproductive tract depend on the infecting IBV strain. For example, the M41, Aust T and QX-like strains are known to cause reproductive tract defects in long-lived chickens [15,17,25], leading to low egg production and quality, whereas IBV strains such as Conn and Iowa609 do not cause reproductive tract abnormalities [15]. The mechanisms that lead certain IBV strains to establish reproductive tract infection are unknown.

However, IBV replication in reproductive mucosa has been documented in several studies [15,61,62]. Depending on the infecting IBV serotype, localization of IBV antigens in the oviduct can vary, and evidence of higher IBV replication has been observed in chickens infected at a younger age when compared to adults [15]. This age difference in IBV replication is reflected in differences in clinical and pathological outcomes in chickens. For example, chickens infected with certain strains of IBV such as Mass, QX-like strain or Aust T at ages of 1–14 days develop cystic oviducts without impaired ovarian functions, which leads to false layer syndrome with no egg production [15,63,64,65]. Such flocks with false layer syndrome do not reach peak production, with a consequence of premature culling [21,63]. Infection with IBV in laying hens can negatively influence egg production, resulting in poor-quality eggs, such as misshapen, miscolored, thin, rough-shelled or shell-less eggs and eggs with watery albumin, meat or blood spots, which can peak at around two weeks post-infection [15,63,66]. In addition, egg production in laying hens can drop by 35–90% [63,67]. Although production bounces back close to normal within nine weeks, there can be a 6–12% decline relative to normal production [63,67]. IBV infection of the reproductive tract of laying hens leads to shorter, hypoglandular oviducts and regressed ovaries [15,66].

Histologically, deciliation of the epithelium and a reduction in the height of the epithelial cells, along with epithelial desquamation and degeneration, are common in infected oviducts between 7 and 21 dpi [66] with occasional follicular destruction and microbleeding [15,68]. Fibroplasia and edema of infected lamina propria are also evident [66]. In contrast to the development of false layers, chickens with patent oviducts were found to have an intact surface epithelium with localized hypoplasia of glandular areas [5]. Later, following around 10 dpi, lymphoid nodules are seen in the oviduct [15]. 

Although the consequences of IBV targeting the reproductive tracts of female chickens is known, little attention has been given to changes corresponding to the reproductive tracts of male chickens, which may cause impacted sperm production, infertility and venereal transmission of IBV [69,70]. Recent studies have found, depending on the infecting IBV strain, that IBV targets the testes and causes low sperm production and infertility [69,71,72]. IBV replication in the efferent duct epithelium and the formation of epididymal stones lead to low sperm production and infertility [69]. Gallardo and colleagues observed IBV replication in cells of the seminiferous tubule (i.e., Sertoli cells) infected with Arkansas (Ark) and Mass IBV strains; moreover, they demonstrated IBV transmission to layers via infected semen, indicating the possibility of venereal transmission of IBV [70]. IBV-induced changes including mononuclear cell and heterophil infiltration, necrosis and microbleeding of testes have been documented [68].

## 6. IBV Infection of the Renal System

The nephropathogenic IBV strains include B1648, Aus T, QX-like, 4/91, Holte and Gray [6,24,25,32,50]. The IBV B1648 strain is known to spread from the respiratory tissues via blood monocytes [50]. Following dissemination, IBV strains could infect ciliated cells of the nephron, including proximal, distal and collecting tubules, depending on the infecting IBV strain [3,32,47,48].

IBV strains that target the kidney have received increased attention due to their higher virulence in young chickens when compared to other IBV strains [13,32,73]. Apart from the general signs associated with IBV infection, nephropathogenic IBV strains result in weight loss, watery droppings, increased water consumption and an increase in the incidence of mortality [6,50]. The nephropathogenic IBV pathogenesis also varies according to the breed of the chickens; for example, the clinicopathological manifestations of nephropathogenic IBV are more severe in Rhode Island Red chickens when compared to White Leghorn chickens [32].

At post-mortem examination, IBV-infected kidneys are pale, discolored and enlarged [32,47]. Urate deposits are also commonly observed with tubular distention [3,32,50]. Histologically, tubules develop degenerative changes, ureters become distended with cellular debris and urate crystals are seen in the tubules; in addition, mononuclear cell recruitment in interstitial tissues in the medulla and cortex has been observed [3,32,50].

## 7. IBV Infection of the Gastrointestinal System

Although IBV has been isolated from cloacal swabs, there is no indication that IBV is transmitted via the fecal–oral route. It is possible that gastrointestinal infection follows respiratory infection, subsequent infection of monocytes and macrophages and the spread of IBV via the blood or lymphatics [50,59]. IBV strains such as QX-like strains, 793/B (4/91) and Moroccan G are known to infect the gastrointestinal tissues, leading to clinical and pathological manifestations [35,74,75,76]. The QX-like strains are capable of targeting the proventriculus and ileum, leading to proventriculitis and, occasionally, diarrhea [56,76]. Moroccan G IBV has also been shown to target the gastrointestinal tissues, such as the esophagus, jejunum, ileum and rectum [74]. Other experimental studies that used Moroccan G IBV indicated that the virus targets the epithelial covering of the tips of villi of the ilium and rectum, leading to atrophy of the villi and desquamation of epithelial cells, with lymphocyte, macrophage and heterophil infiltration in the mucosa [3,35]. IBV 793/B (i.e., 4/91) has also been shown to replicate in the esophagus and ileum, leading to enteritis in young broiler chickens [35].

## 8. Impact of IBV on the Muscular System

In the early 1990s, broiler chickens infected with IBV strain 793/B were found to develop bilateral pectoral myopathy [34]. The disease was characterized by edema, due to a gelatinous material, followed by facial hemorrhage and mild separation of muscle fibers [34]. Although bilateral myopathy could not be reproduced with IBV 793/B, mild gross changes were observed with no indication of histological changes or muscle damage [35]. Further, a study that collected samples with bilateral pectoral myopathy from a slaughter plant in Brazil could not establish an association of this condition with IBV infection, although IBV was detected in muscle tissues using molecular techniques [77]. Consequently, the muscle lesions were suggested to be caused by type III hypersensitivity involving the deposition of immune complexes in the capillary walls of pectoral muscles, rather than lesions induced by viral replication [35].

## 9. IBV Persistence

Depending on the IBV strain, the virus can persist in the tissues of chickens for an extended period [40,74,78]. For example, IBV (i.e., Aust T strain) can persist, particularly in the cecal tonsils and kidney, for more than seven months [40]. The Mass IBV strain can persist in the cecal tonsils, spleen and kidney for about a month [78]. Although the period of IBV persistence is influenced by the age at infection [42], the length of persistence does not depend on the systemic anti-IBV antibody concentration [4]. The implications of IBV persistence in chickens are twofold. First, persistently infected chickens are a source of infection for naïve chickens [4]. Second, persistent IBV infection promotes viral evolution [4].

## 10. Immunosuppressive Effects of IBV

**Figure 3:**
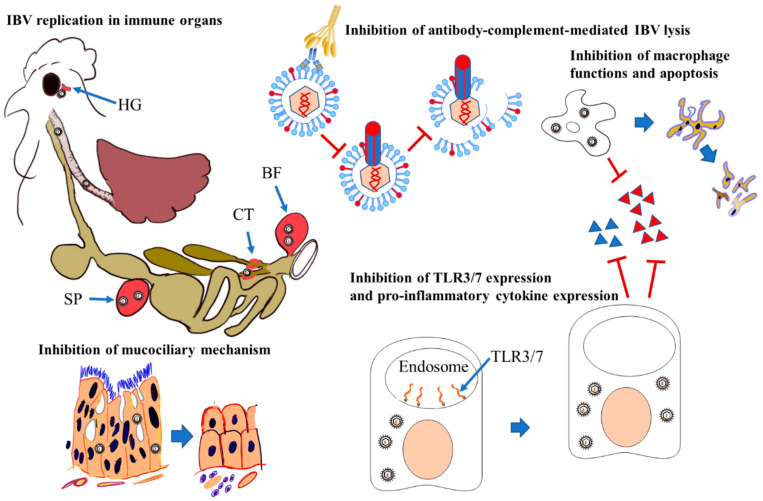
Immune evasion mechanisms of IBV. IBV replicates in multiple immune organs, such as the bursa of Fabricius (BF), spleen (SP), cecal tonsils (CT) and Harderian gland (HG), enabling it to persist in the host for a longer period. Epithelial cells are the primary target sites of IBV replication, which results in deciliation and destruction, leading to inhibition of the mucociliary escalator mechanism. Certain strains of IBV can inhibit the expression of pathogen recognition receptors such as toll-like receptors (TLRs) or their signaling pathway, leading to reduced expression of proinflammatory cytokines, which eventually interferes with the innate immune response and induction of the adaptive immune response. IBV is also capable of replicating in respiratory tract macrophages, inhibiting their functions and inducing apoptosis. IBV is capable of incorporating CD59 molecules into its envelop during egress from the host cells, shielding it against lysis via complement- and antibody-dependent mechanisms.

The IBV-induced potential immune-suppressive mechanisms are summarized in Figure 3. IBV strains 4/91, QX-like, Strain G and Mass infect various immune organs, such as the cecal tonsils [35], spleen [45], Harderian gland and bursa of Fabricius [3,79]. It is not known whether the tropism of IBV for these immune organs depends on the virulence of the infecting IBV strain or whether IBV replication in these immune organs impacts immune functions. It is very well documented that avian viruses that replicate in immune organs, such as Marek’s disease virus [80], chicken anemia virus [81,82] and infectious bursal disease virus [83], are immunosuppressive, impacting vaccine-mediated immune response and resulting in secondary bacterial infections [84]. However, recent investigations provided molecular and cellular evidence that IBV, in fact, directly interferes with the host’s innate response at various levels, potentially impacting the elicitation of adaptive host response. The toll-like receptors (TLRs) 3 and 7 are innate receptors and have a role in detecting IBV-associated molecular patterns, such as double-stranded (ds) and single-stranded (ss) ribonucleic acid (RNA), respectively [85]. Certain Brazilian strains of IBV are capable of inhibiting TLR7 signaling, leading to decreased proinflammatory cytokines and decreased mRNA expression linked to the development of cell-mediated immune response, leading to increased pathology in the kidney [86]. Similarly, a Conn strain of IBV has been shown to downregulate mRNA expression of TLR3, interleukin (IL)-1β and interferon gamma (IFN-γ), leading to increased IBV genome accumulation and more severe pathology in the respiratory tissues [87].

One of the immune cell types that bridges innate and adaptive host responses is the macrophages, and the available data show that certain IBV serotypes (i.e., Mass and Conn) target respiratory tract macrophages and replicate within them, thus leading to a productive infection [59,88]. Although the impact of immune cell targeting of IBV has not been studied completely, IBV replication in macrophages could decrease type 1 IFNs activity [59], similar to IBV’s ability to hinder type 1 IFNs response in epithelial and fibroblast cells [89]. Type 1 IFNs are the main antiviral molecules synthesized in the host in response to viral replication, and, previously, it has been shown that IBV is sensitive to the antiviral activity of type 1 IFNs given as a treatment to prevent IB in chickens [90]. Although it is not known how IBV inhibits the function of type 1 IFNs in macrophages, one potential explanation is that the accessory proteins of IBV could play a role in this immune evasion strategy. In agreement with this view, the ability of IBV accessary proteins 3a and 5b to interfere with type 1 IFNs production in target cells other than macrophages has been shown [89,91]. Other than this interference with the production of type 1 IFNs, IBV is capable of inhibiting the downstream signaling of type 1 IFNs, minimizing the expression of interferon-stimulating genes (ISGs) by preventing the functioning of signal transducer and activator of transcription 1 (STAT1) [89]. Another implication of IBV replication in macrophages is the destruction of macrophages due to apoptosis. IBV could induce programmed cell death in avian macrophages via intrinsic and extrinsic routes involved in apoptosis [88]. Although the mechanisms of the destruction of macrophages by IBV require further investigation, evidence has shown that macrophage numbers increase at 24 h and then decline in the trachea and lungs in response to IBV infection [87]. It is important to understand whether this decline is related to the apoptosis of IBV-infected macrophages.

The complement system functions as a part of the innate arm of the immune response and also plays a role in adaptive immune functions. The components of the complement pathway aid in the host immune response via complement- and antibody-mediated lysis of viruses [92,93,94,95]. Since host cells are shielded from lysis via this strategy due to the expression of CD59 in the host cell membrane, IBV is capable of incorporating CD59 into its envelop during exit from the host cells, negating lysis via complement- and antibody-dependent mechanisms [96]. Previous studies showed that IBV is vulnerable to attack by this strategy [97].

IBV replication impacting the respiratory tract brings forth a defective clearance mechanism by the deciliation of the respiratory epithelium, thereby increasing the vulnerability of respiratory surfaces to secondary bacterial infections [98]. The co-infection of respiratory pathogens with IBV causes complications and worsens the clinical and pathological manifestations [18,99]. The infection of chickens with *Mycoplasma gallisepticum* followed by IBV infection can result in coryza, tracheitis and airsacculitis in the host [100]. In addition, secondary infections with pathogenic *Escherichia coli* can lead to prominent lesions in respiratory surfaces, pericarditis and death [18]. In addition, the co-infection of *Haemophilus paragallinarum* with IBV could result in severe lesion development and increased mortality rates [101].

## 11. Vaccine Mediated IB Control

Protection against IB is mediated by both antibody- and cell-mediated immune responses [102,103]. Although the antibody-mediated immune response predominantly depends on a response to the IBV S1 protein, the main cell-mediated response, CD8+ cytotoxic T cell response is elicited by the IBV N protein [103]. Following IBV infection, memory B [104] and T cells [105] are formed and are present in peripheral blood and the spleen. Following IB vaccination, the number of B and T cells increases in the harderian gland [106]. Increased recruitments of CD4+ and CD8+ T cells in the trachea have also been shown following application of live IB vaccines [107].

Vaccination is the primary choice for the control of IB in the poultry industry. The chickens receive multiple IB vaccinations, and the frequency of vaccination depends on the expected production life of the chicken. For broilers, day-old vaccination in the hatchery is commonly practiced followed by a booster vaccination at 2–3 weeks of age [99]. Multiple vaccinations with live attenuated vaccines are usually given to layers and breeders followed by a killed vaccine just before the onset of the laying period [108,109]. The live attenuated vaccines are administered via drinking water, eye drop, or coarse spray and inactivated vaccines are given parenterally. For example, in Eastern Canada, the layer and breeder pullets are vaccinated with various combinations of live attenuated IB vaccines starting at one day of age and then, at two, five and nine weeks followed by an inactivated IB vaccine given at fourteen weeks of age. During the lay, the chickens are not vaccinated. The goal of such layer and breeder vaccination strategies is to ensure the transfer of maternal antibodies to the offspring [110] as well as provide the extended protection during the lay [111,112]. 

However, vaccination of day-old chickens is controversial for three reasons. First, maternal antibodies could be protective against potential IBV infection during the first few days of life while early vaccination could enhance the decay of the maternal antibodies [110]. Second, early vaccination (i.e., Day 1 of age) induces poor B cell and T cell responses when compared to later vaccination (i.e., Day 7 of age) [106]. Third, IB vaccination at the day of age can induce severe vaccine reactions [113]. Despite these reasons, live attenuated IB vaccination in the hatchery can be critical for the prevention of severe respiratory illness and cystic lesions in the oviduct that results from very early exposure to virulent IBVs [65,114].

Since the introduction of vaccination against IB using live attenuated (Mass serotype) vaccines, different vaccine serotypes such as 4/91, Ark, Conn, D274 and D1466 have been developed and made available commercially [115]. Based on the prevalence of the various IBV serotypes, and the availability of licensed vaccines in a geographical area, different areas within countries use different combinations of live attenuated and inactivated vaccines. In certain regions in Europe, vaccines against Mass and 4/91 serotypes are widely used, while, in certain states within USA, vaccines against Mass, Ark and Conn serotypes are extensively used [108]. The serological response to IBV is mainly induced by the S1 protein [116,117] and, consequently, cross-protection induced by IB vaccines against heterologous strains varies widely. The study of Cook and colleagues indicated that the use of more than one vaccine serotype can produce better cross-protection against challenge with heterologous IBV serotypes [118]. Therefore, priming with more than one heterologous live vaccines and boosting with inactivated or live attenuated vaccines are practiced particularly by layer and breeder industries [65]. It has also been shown that vaccination protocols that involve more than one serotype induced better immune cell recruitment in the respiratory mucosa when compared to vaccination with a single serotype vaccine [107].

Although control of IB relies on vaccination [113], several limitations of IB vaccination have been observed. The steady increase of IBV variants leading to frequent outbreaks in vaccinated flocks has become a concern increasingly [99,119,120]. In addition, attenuated IBV strains used for vaccination can spread among individual birds within flocks [121], and changes in virulence during bird-to-bird passage can lead to production problems [120,122]. Coinfection of host cells with live attenuated IB vaccines and wild type IBV can also result in genomic recombination contributing to the virus evolution [11,123] as well as mutations that can occur under the effect of immune pressure [124,125,126]. Although the global poultry industry practices hatchery vaccination against IB, Day 1 may not be the optimum time for vaccination in terms of inducing systemic and mucosal immune responses against IB [106] because of the developing immune system [127]. The availability of a limited number of licensed vaccines to choose in a geographical area is also a constraint. For example, in Canada, various IBV variants including Mass, Conn, 4/91, CA1737 and DMV/1639 [11,23,128,129,130] are circulating in poultry flocks and only live attenuated vaccines developed against Mass and Conn serotypes and inactivated vaccines developed against Mass and Ark serotypes are available for optimizing on farm IB vaccination strategies. 

Given the limited efficacy of existing IB vaccination strategies, it is critical to establish an IBV surveillance system that characterizes the different IBV strains circulating in various geographical areas and that we understand the antigenic and/or genetic similarities between the circulating IBVs and the available IB vaccines. These data will lead to the optimization of IB vaccination strategies to prevent vaccine breaks. If the existing vaccines are not useful in optimizing vaccination strategies, it would also worth developing autogenous vaccines (inactivated) using characterized unique IBV isolates prevalent in a given geographical area in order to include in the existing vaccination regimes. Given the available scientific evidence against IB vaccination at the hatchery [106,110], it may also be appropriate to postpone the first IB vaccination to a barn vaccination done at seven days of age relying on maternal antibody response to protect the chickens during the first week of life [110]. There are numerous issues surrounding the use of live attenuated IB vaccines [11,99,119,120,121,122,123,124,125,126], and relying on inactivated vaccines can be an option, but they inherently lack the ability to induce mucosal immune response, which is critical for the control of IB [131,132,133]. It is possible that inactivated vaccines can be used for the induction of mucosal immune response when combined with various nanoparticles [132].

## 12. Conclusions

After decades of research into IBV and numerous studies on vaccine efficacy for the control of IB, this disease is still a major economic concern globally. Although many studies have been conducted on IBV pathogenesis, there is little specific information on IBV receptors that determine macrophage tropism and tissue tropism, mechanisms that allow IBV dissemination from the respiratory tract to secondary tissues, IBV persistence in the cecal tonsils and kidney and the immunopathogenesis and immunosuppressive mechanisms of different strains of IBV. Novel and sensitive assays and other necessary tools are now available for in-depth investigations of the mechanisms involved in these pathogenesis events. Given the concern of vaccine breaks and the emergence of heterogeneous IBV strains, investigations leading to an in-depth understanding of the pathogenesis of IBV are necessary.

Studies of host–IBV interaction lead to the understanding of tropism of IBV for various body systems, severity of lesions produced in each of these tissues and ways the virus is shed to the environment [3,51,64,134]. For example, certain IBV variants impact tissues such as kidney and reproductive tract in addition to the respiratory tract [13,23,32,33]. We are not aware if the current vaccination protocols induce adequate mucosal immune responses in each of these tissues to minimize consequences of IBV replication. It is critical to optimize IB vaccination strategies that induce adequate immune responses in target body systems of these IBV variants minimizing the impact. 

In addition to secondary bacterial infections, vaccine induced immune response to any pathogen can be impacted by immune suppression induced by IBV. It is necessary to determine if IBV induced immune suppression impact the immune responses intended to be generated by a variety of vaccines used in chickens [127]. Another area worth investigating is whether IB vaccines are immunosuppressive similar to wild type IBV.
